# The severity of retinal pathology in homozygous *Crb1^rd8/rd8^* mice is dependent on additional genetic factors

**DOI:** 10.1093/hmg/ddu424

**Published:** 2014-08-21

**Authors:** Ulrich F.O. Luhmann, Livia S. Carvalho, Sophia-Martha kleine Holthaus, Jill A. Cowing, Simon Greenaway, Colin J. Chu, Philipp Herrmann, Alexander J. Smith, Peter M.G. Munro, Paul Potter, James W.B. Bainbridge, Robin R. Ali

**Affiliations:** 1 Department of Genetics and; 2 Imaging Unit, UCL Institute of Ophthalmology, London EC1V 9EL, UK; 3 MRC Laboratory for Molecular Cell Biology, University College London, London WC1E 6BT, UK; 4 Mammalian Genetics Unit, MRC Harwell, Oxfordshire OX11 ORD, UK and; 5 NIHR Biomedical Research Centre at Moorfields Eye Hospital NHS Foundation Trust and UCL Institute of Ophthalmology, London EC1V 2PD, UK

## Abstract

Understanding phenotype–genotype correlations in retinal degeneration is a major challenge. Mutations in *CRB1* lead to a spectrum of autosomal recessive retinal dystrophies with variable phenotypes suggesting the influence of modifying factors. To establish the contribution of the genetic background to phenotypic variability associated with the *Crb1^rd8/rd8^* mutation, we compared the retinal pathology of *Crb1^rd8/rd8^/J* inbred mice with that of two *Crb1^rd8/rd8^* lines backcrossed with *C57BL/6JOlaHsd* mice. Topical endoscopic fundal imaging and scanning laser ophthalmoscopy fundus images of all three *Crb1^rd8/rd8^* lines showed a significant increase in the number of inferior retinal lesions that was strikingly variable between the lines. Optical coherence tomography, semithin, ultrastructural morphology and assessment of inflammatory and vascular marker by immunohistochemistry and quantitative reverse transcriptase-polymerase chain reaction revealed that the lesions were associated with photoreceptor death, Müller and microglia activation and telangiectasia-like vascular remodelling—features that were stable in the inbred, variable in the second, but virtually absent in the third *Crb1^rd8/rd8^* line, even at 12 months of age. This suggests that the *Crb1^rd8/rd8^* mutation is necessary, but not sufficient for the development of these degenerative features. By whole-genome SNP analysis of the genotype–phenotype correlation, a candidate region on chromosome 15 was identified. This may carry one or more genetic modifiers for the manifestation of the retinal pathology associated with mutations in *Crb1.* This study also provides insight into the nature of the retinal vascular lesions that likely represent a clinical correlate for the formation of retinal telangiectasia or Coats-like vasculopathy in patients with *CRB1* mutations that are thought to depend on such genetic modifiers.

## INTRODUCTION

Understanding the genotype–phenotype correlation in retinal degenerations remains a major challenge even for monogenetic diseases. Although the causality of many primary mutations has been convincingly established, the clinical manifestation of even identical mutations can be highly variable. This may be due to additional genetic polymorphisms in the same gene (allelic heterogeneity) or in additional genes (genetic modifiers) as well as environmental factors. The combination of all these factors in a patient may influence age of onset, progression and severity of disease as well as the development of particular phenotypic features associated with a primary mutation (1,2).

One example of mutations in a gene that lead to a highly variable spectrum of clinical phenotypes is the *CRB1* gene (OMIM #604210). They typically cause a spectrum of autosomal recessive (ar) rod-cone dystrophies that ranges from retinitis pigmentosa (RP12) (3) to Lebers congenital amaurosis (LCA) (4,5). Both arRP- and arLCA-patients that carry *CRB1* mutations may show additional specific features in fundus images such as preservation of para-arteriolar retinal pigment epithelium and retinal telangiectasia with exudates (also called Coats-like vasculopathy) (3–7). So far no genotype–phenotype correlation with mutations in *CRB1* for any of these clinical features has been established, except that *CRB1* null-mutations may be over-represented in LCA cases (5–7). This wide range of clinical characteristics in patients with *CRB1* mutations suggests that additional genetic and environmental modifiers influence the development of the disease (5,6,8).

CRB1 is a member of the highly conserved CRB protein family that in mammals comprises two additional members, CRB2 and CRB3 (9,10). While expression of CRB1 is restricted to the retina and the brain (3), CRB2 and CRB3 show a wider range of tissue expression (11,12).

In the retina, CRB1 is part of the CRB/Crb complex, which is localized at the outer limiting membrane (OLM) where it sits just apically to the adhesion junctions (AJ) that connect photoreceptors with each other and with Müller glia cells (10). The CRB/Crb complex and Crb1 in particular seem to regulate the polarity of these cells and appear crucial for maintaining stratification of the retina (10,13–16).

Two mouse models with different mutant *Crb1* alleles exist (13,16). The *Crb1^rd8^* allele carries a single base pair deletion of a cytosine in exon 9 (*Crb1*. 3841delC) that causes a frame shift and premature stop codon resulting in a predicted truncated protein that only consists of the N-terminal extracellular domain (17). The *Crb1* null allele instead leads to complete ablation of Crb1 protein expression by genetic targeting of the *Crb1* gene sequence containing the upstream promoter, exon 1 with the start codon and part of intron 1 (13). Consistent with the autosomal recessive trait of arRP and arLCA, only homozygous mice for each of the two alleles show degenerative changes in the retina (13,16). *Crb1^rd8/rd8^* mice exhibited a prominent focal inferior retinal degeneration characterized by the formation of local retinal folds, pseudorosettes, photoreceptor loss and retinal thinning with earliest signs of degeneration at 2 weeks of age (16). *Crb1^−/−^* mice also develop an inferior retinal degeneration, but their degenerative features are less prominent and frequent, comprising of small localized photoreceptor displacements, half rosettes and loss of OLM in affected areas and are only consistently seen from 3 months of age onwards (13,15,16). This variability in age of onset and severity of the retinal degeneration between lines carrying different alleles suggests that either the allelic heterogeneity contributes to the phenotypic differences or that, similar to humans, the phenotype in mice is highly variable and may be influenced by genetic and environmental factors.

Many other inbred knockout mouse and ES cell lines also carry the *Crb1^rd8/rd8^* mutation in a homozygous state (18). This mutation therefore has likely confounded the phenotypic description and subsequent conclusions drawn from many mouse models for retinal disease. One recent example is an early onset retinal degeneration seen in *Ccl2^−/−^/Cx3cr1^−/−^* double knockout mice that turned out to be dependent on the homozygous *Crb1^rd8/rd8^* allele instead on a previously proposed synergistic effect of the combined knockout of both chemokines (19). To prevent such misinterpretation due to the unnoticed presence of the *Crb1^rd8/rd8^* mutation in the future, we here aim to evaluate the range and variability of phenotypic features associated with this mutation. Therefore, we compared the retinal pathology of three homozygous *Crb1^rd8/rd8^* mouse lines that were of different genetic origin but were raised in an identical environment. We describe primary consequences of the *Crb1^rd8/rd8^* mutation, identify secondary phenotypic features that did not manifest in all *Crb1^rd8/rd8^* lines and identify a candidate region on chromosome 15 that may carry additional genetic factors that determine the severity of the retinal pathology caused by mutations in *Crb1*.

## RESULTS

**Figure 1.**
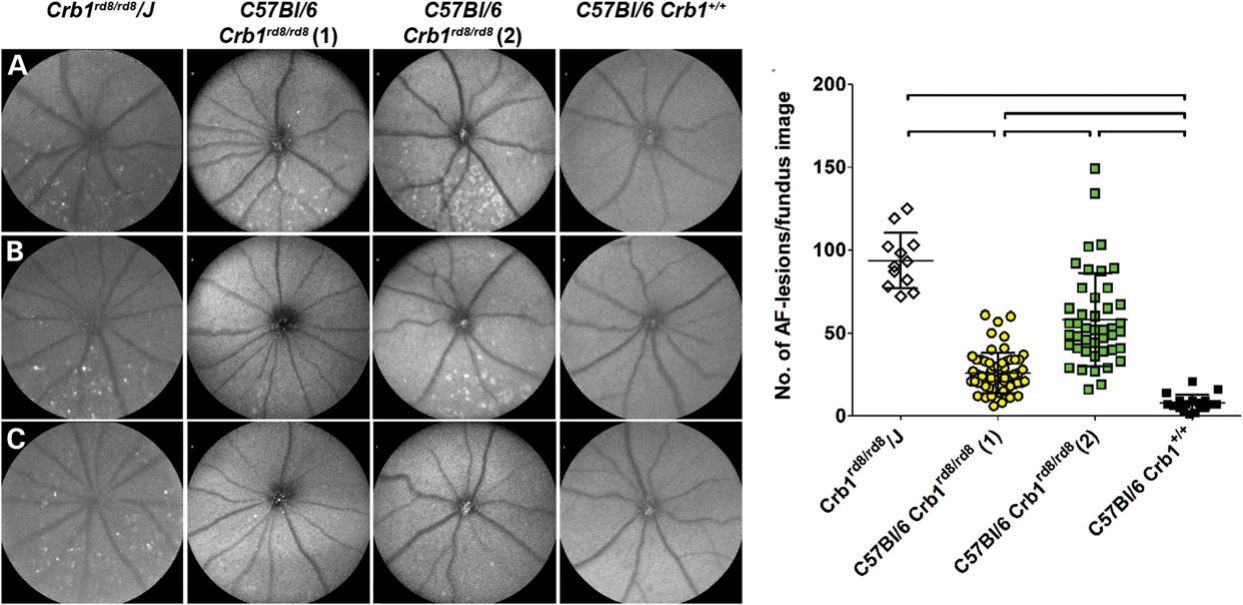
Variable manifestation of the inferior retinal degeneration in different *Crb1^rd8/rd8^* mouse lines at 2 months of age. Qualitative and quantitative *in vivo* phenotyping by AF-SLO of three *Crb1^rd8/rd8^* mouse lines and *C57BL/6J Crb1^+/+^* control mice revealed the variable manifestation of large irregular lesions in the inferior retina. Three representative AF-SLO fundus images for each of the *Crb1^rd8/rd8^* lines are shown (**A**–**C**, *Crb1^rd8/rd8^/J*; **D**–**F**: *C57BL/6 Crb1^rd8/rd8^* (1); **G**–**I**: *C57BL/6 Crb1^rd8/rd8^* (2); **J**–**L**: *C57BL/6J Crb1^+/+^*). M: quantification of the number of lesions per fundus image differed significantly in variance (Bartlett's test for equal variances: *P* < 0.0001) and in number of lesions between *Crb1^rd8/rd8^* mouse lines. Black lines indicate significant differences with *P* < 0.0001 using a one-way ANOVA with Tukey's multiple comparison test.

To establish the degree of phenotypic variability in homozygous *Crb1^rd8/rd8^* mice, we analysed three different *Crb1^rd8/rd8^* mouse lines at 2 months of age using autofluorescent scanning laser ophthalmoscopy (AF-SLO; Fig. 1). All *Crb1^rd8/rd8^* lines showed significantly higher number of autofluorescent lesions in the inferior retina than wild-type mice (Fig. 1). However, a striking variability in the manifestation of this phenotype was observed between lines. While the genetically independent *Crb1^rd8/rd8^/J* inbred line showed high and stable number of characteristic large irregular lesions (Fig. 1A–C), significantly fewer lesions were observed in the two closely related lines [Fig. 1M, *C57BL/6 Crb1^rd8/rd8^* (1) and (2)]. Autofluorescent lesions in *C57BL/6* C*rb1^rd8/rd8^* (1) mice were small, weak, reduced in number and located more inferiorly (Fig. 1D–F), while typical large lesions were seen in *C57BL/6/*C*rb1^rd8/rd8^* (2) mice, but with a high degree of variability (Fig. 1G–I and M).

### Large irregular inferior lesions that correspond to localized displacement of photoreceptors are not observed in all homozygous *Crb1^rd8/rd8^* mice

**Figure 2.**
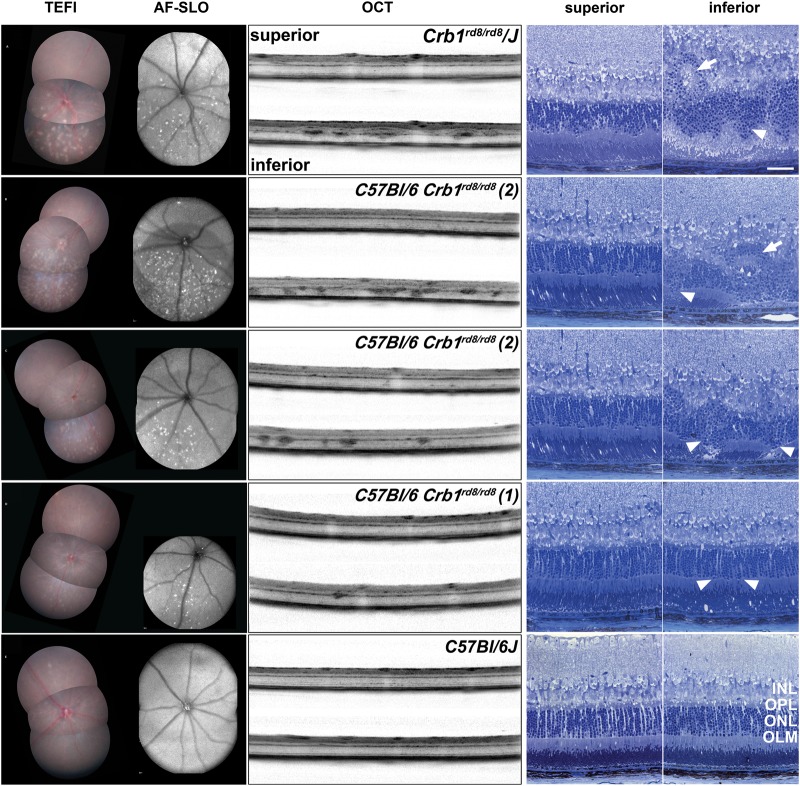
Retinal pathology of different *Crb1^rd8/rd8^* mouse lines illustrated by corresponding TEFI, AF-SLO fundus images and sagittal OCT and semithin sections at 2 months of age. (**A**–**D**) Example images of the different modalities including superior and inferior images from homozygous C*rb1^rd8/rd8^* mice from the respective lines. (A) *Crb1^rd8/rd8^/J*, (B, C) *C57BL/6/*C*rb1^rd8/rd8^* (2), (D) *C57BL/6/*C*rb1^rd8/rd8^* (1) and (**E**): *C57BL/6J* wild-type mice (C*rb1^+/+^*). White arrow heads indicate lesions affecting the structure of the ONL and the integrity of the OLM. White arrows indicate rosette formation observed in mice with severe manifestations of the phenotype. These lesions are preferentially located in the inferior retina and correspond well with autofluorescent lesions in AF-SLO fundus images and with white/opaque lesions in the fundus images obtained by TEFI. INL, inner nuclear layer; OPL, outer plexiform layer; ONL, outer nuclear layer; OLM, outer limiting membrane. Scale bar for semithin images: 50 μm.

Topical endoscopic fundal imaging (TEFI), AF-SLO, optical coherence tomography (OCT) and semithin histology revealed that this pathology was mainly confined to the inferior retina of *Crb1^rd8/rd8^* mice (Fig. 2). White/opaque and autofluorescent fundus lesions correlated well with the position of lesions in OCT images at the outer plexiform layer (OPL) and in the outer nuclear layer (ONL) (Fig. 2A–C). Corresponding rosette formation (Fig. 2A and B, white arrows) and drop out and disruption of photoreceptor columns (Fig. 2A–C, arrow heads) were observed in sagittal semithin sections of prominently affected *Crb1^rd8/rd8^* mice. However, not all *Crb1^rd8/rd8^* mice showed these inferior lesions by TEFI or AF-SLO. In particular, in the *C57BL/6/*C*rb1^rd8/rd8^* (1) mice only small inferior lesions were visible (Fig. 2D) that were very difficult to identify in OCT and semithin sections (Fig. 2D) since these appeared to be very similar to those from wild-type mice (Fig. 2E).

### The *Crb1^rd8/rd8^* mutation leads to reduced *Crb1* mRNA levels and absence of the CRB1 protein at the OLM

**Figure 3.**
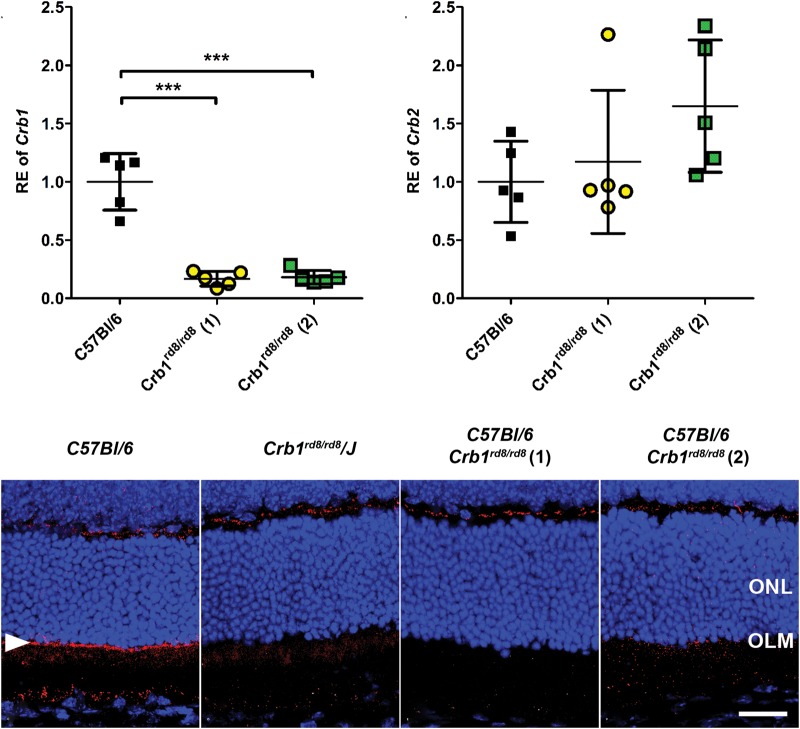
The *Crb1^rd8/rd8^* mutation leads to reduced *Crb1* mRNA levels and the absence of the Crb1 protein at the OLM. mRNA expression analysis for *Crb1* (**A**) and *Crb2* (**B**) by quantitative real-time RT-PCR and protein localization of Crb1 by immunohistochemistry (**C**–**F**) in retinas of wild-type (*Crb1^+/+^*) and homozygous *Crb1^rd8/rd8^* mice from the different lines at 8 weeks of age. (A) Quantitative real-time PCR detecting the exon 8 to mutant exon 9 boundary of *Crb1* revealed similar reduction of mutant *Crb1* transcripts in all homozygous *Crb1^rd8/rd8^* mice compared with wild type. Asterisks indicate a statistically significant difference at *P* < 0.001 using one-way ANOVA with Tukey's multiple comparison test. (B) No significant compensatory increase of *Crb2* mRNA levels in either of the *Crb1^rd8/rd8^* lines (*P* = 0.1693; one-way ANOVA with Tukey's multiple comparison test). (C–F) Immunohistochemistry for Crb1 on sagittal retinal sections shows a specific signal (red) for Crb1 at the level of the OLM (white arrow head) in the wild type (C), which was not detected in all other homozygous *Crb1^rd8/rd8^* mouse lines (D–F). ONL, outer nuclear layer. Scale bar: 25 µm.

To further understand whether the *Crb1*^rd8^ allele, possibly by altered expression of the predicted truncated CRB1 protein (16), contributes to the phenotypic variability in different strains and whether expression of *Crb2* may be altered in a compensatory way, we quantified *Crb1* and *Crb2* transcript levels relative to wild type in weakly and severely affected *Crb1^rd8/rd8^* mice (Fig. 3). We also evaluated whether the genetic background of different inbred mouse strains influences the expression level of *Crb1* (Supplementary Material, Fig. S1). *Crb1* transcripts were significantly reduced to ∼20% of wild type in all *Crb1^rd8/rd8^* mice, independent of the severity of their phenotypes (Fig. 3A). No significant changes in *Crb2* transcript levels were observed, although subtle effects cannot be excluded (Fig. 3B). Although *Crb1* transcript levels were significantly influenced by different genetic backgrounds, the influence of the homozygous *Crb1^rd8/rd8^* mutation on *Crb1* transcript levels was always much more pronounced (Supplementary Material, Fig. S1). Consistent with these data, immunohistochemistry using a C-terminal antibody for Crb1 revealed a weak specific signal across the whole superior and inferior OLM in wild-type retina (OLM; Fig. 3C, white arrow head), but not in any retina from *Crb1^rd8/rd8^* mice (Fig. 3D–F).

### The lack of *Crb1* leads to a variable reduction of number of adhesions junctions at the OLM across the whole retina

**Figure 4.**
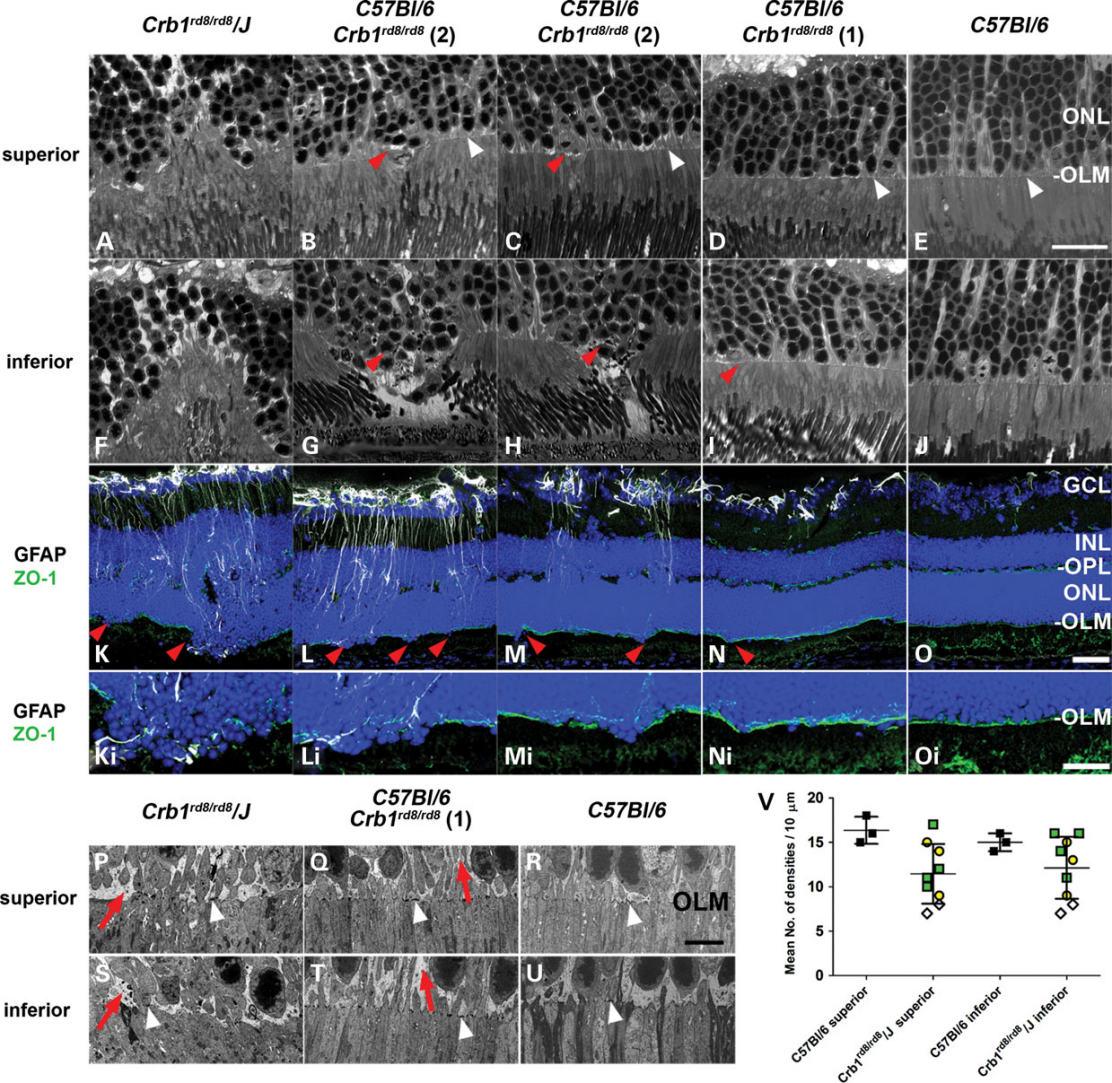
Assessment of OLM integrity and associated Müller glia activation in the superior and inferior retina of different C*rb1^rd8/rd8^* mouse lines. Corresponding confocal bright field images of the superior (**A**–**E**) and inferior (**F**–**J**) OLM (white arrow heads) in retinas of animals from respective lines. Scale bar: 20 µm. (**K**–**O**) Corresponding images of the inferior retina obtained by immunohistochemistry for glial fibrillary acidic protein (GFAP, white, activated Müller glia and astrocytes), the tight junction protein ZO-1 (green) and 4′,6-diamidino-2-phenylindole (DAPI, blue, nuclei) on sagittal retinal cryo-sections. Scale bar: 50 µm. (**K**_i_–**O**_i_) Magnified views of the inferior disrupted OLM. Scale bar: 25 µm. Red arrow heads indicate predominant disruption of the OLM in areas of the inferior retina where photoreceptor disruption in the ONL is associated with localized Müller cell activation (white, GFAP^+^ processes of Müller glia cells). (**P**–**U**) Assessment of integrity of the OLM by TEM. Representative TEM images with electron dense adhesions plaques (white arrow heads) and Müller glia processes (red arrows) from the superior and inferior central retina of *Crb1^rd8/rd8^/J* mice, *C57BL/6 Crb1^rd8/rd8^* (line 1) and wild-type mice. Scale bar: 5 µm. (**V**) Quantification of number of electron dense AJ normalized to the assessed distance of OLM and expressed as number of densities per 10 µm. Different colours and shapes represent the origin of individual wild-type or *Crb1^rd8/rd8^* mice (black square: *C57BL/6J* controls, yellow circle: *C57BL/6J Crb1^rd8/rd8^* (1), green square: *C57BL/6J Crb1^rd8/rd8^* (2) and open diamond: *Crb1^rd8/rd8^/J*). Overall, the number of AJ per 10 µm shows a similar range of variable reduction in the superior and inferior retina of *Crb1^rd8/rd8^* mice compared with wild-type mice, although this feature was not consistent in all *Crb1* deficient mice.

To better understand how the loss of the evenly distributed CRB1 protein at the OLM might lead to a preferential degeneration in the inferior retina and to see whether all *Crb1^rd8/rd8^* homozygous mice are similarly affected, we evaluated the integrity of the OLM in the three differentially affected *Crb1^rd8/rd8^* lines (Fig. 4). Confocal evaluation on semithin sections across the whole retina suggested that the OLM of *C57BL/6 Crb1^rd8/rd8^* (1) mice is macroscopically similar to that of wild-type mice (white arrow heads; Fig. 4D versus E and I versus J). Also the tight junction protein ZO-1 is evenly distributed along the whole OLM in all *Crb1^rd8/rd8^* and wild-type mice (Fig. 4K–O and Ki–Oi). ZO-1 labelling only became disrupted in the inferior retina if photoreceptor columns were disorganized (red arrow heads, Fig. 4F–J), photoreceptor nuclei dropped out of the ONL (red arrow heads, Fig. 4F–J) and localized Müller glia became activated (Fig. 4K–M). All these features were barely detectable in *C57BL/6 Crb1^rd8/rd8^* (1) mice (Fig. 4N and Ni, red arrowhead) and difficult to distinguish from observations in wild-type mice (Fig. 4E and J and O and Oi).

Quantitative ultrastructural analyses revealed a variable reduction of adhesions junctions at the OLM from 16 ± 1 AJ's (range: 14–18) per 10 µm in wild-type mice to 12 ± 3 per 10 µm (range: 7–14) in the superior and inferior retina of *Crb1^rd8/rd8^* mice (Fig. 4V). This reduction was not consistently observed in all homozygous animals from both *C57BL/6* C*rb1^rd8/rd8^* (1 and 2) lines. Also the expansion of the glial component at the OLM seems similar at the inferior and superior OLM within each line, but different between them (Fig. 4P, Q, S and T, red arrows). Despite these line specific differences, both AJ reduction and glial expansion seem to be primary consequences of the loss of Crb1 that contributes to an even weakening of the OLM across the superior and inferior retina.

### Dying photoreceptors and associated local Müller and microglia activation in the inferior retina were not detected in weakly affected *Crb1^rd8/rd8^* mice

**Figure 5.**
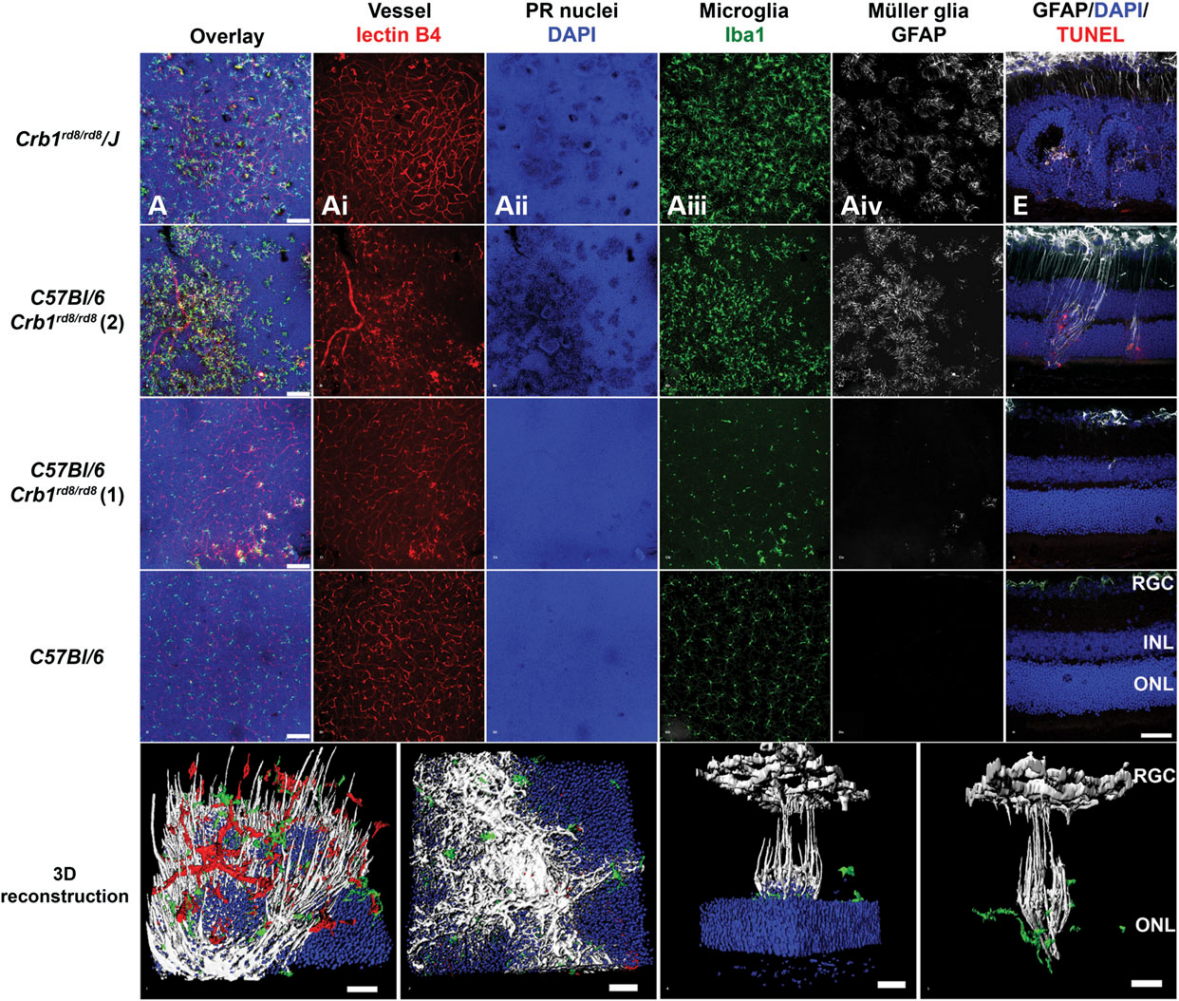
Localized Müller and microglia activation occurs predominantly in the inferior retina and is associated with dying photoreceptors and vascular changes. (**A**–**D**) Overlay of projection images of confocal *z*-stacks of 30–40 µm height located around the outer plexiform layer (OPL) of the central inferior retina of respective *Crb1^rd8/rd8^* mouse lines and wild-type controls at 2 months of age. For orientation, the deep retinal vascular plexus was labelled by Isolectin B4 (red, **A**_i_, **B**_i_, **C**_i_, **D**_i_) and the photoreceptor nuclei of the ONL by DAPI (blue, **A**_ii_, **B**_ii_, **C**_ii_, **D**_ii_). Microglia and Müller glia activation in the inner retina was assessed by Iba1 (green, **A**_iii_, **B**_iii_, **C**_iii_, **D**_iii_) and GFAP (white, **A**_iv_, **B**_iv_, **C**_iv_, **D**_iv_) labelling. Scale bar: 75 µm. (**E**–**H**) Photoreceptor death in the retina was assessed qualitatively by TUNEL^+^ labelling (red) on superior to inferior oriented sagittal retinal sections and co-labelled with GFAP (white) for Müller cell activation. Images taken from the inferior central retina are shown. Localized GFAP activation was closely associated with TUNEL^+^ (red) photoreceptor nuclei (blue). Scale bar: 50 µm. (**I**–**L**) 3D reconstruction using Imaris software showing lectinB_4_^+^ endothelial cells (red), Iba1^+^ microglia (green), DAPI-labelled photoreceptor nuclei in the ONL photoreceptor (blue) and GFAP^+^ Müller cells (white). (I) View at the OPL towards the ONL. (J) Pronounced gliotic scar located at the outer surface of the ONL facing the subretinal space. (J–I) Scale bar: 20 µm. 3D rotated side views of a single inferior lesion with (K) and without (L) representation of the ONL. Columns of photoreceptor nuclei that are elevated from the ONL (K) are surrounded by activated GFAP^+^ Müller cell processes and Iba1^+^ microglia (K, L) suggesting a localized glial response around these abnormally arranged photoreceptors. At the retinal ganglion cell layer, GFAP also labels astrocytes next to Müller cell processes. (K, L) Scale bar: 200 µm.

The prominent degenerative changes in the inferior retina of some *Crb1^rd8/rd8^* mice lead us to further investigate an association between photoreceptor death and Müller and microglia activation (Fig. 5). Prominent loss of DAPI positive signal on retinal flat mounts (Fig. 5A, B, A_ii_ and B_ii_) and TUNEL^+^ photoreceptor nuclei in sections (Fig. 5E) are consistent with the darker labelling of photoreceptor nuclei (denser chromatin, pyknotic cell nuclei) and inner segments observed at the base of the whorls in semithin histology (e.g. Fig. 2A and C) and all together indicated loss of photoreceptors at the ONL in inbred *Crb1^rd8/rd8^/J* and *C57BL/6 Crb1^rd8/rd8^* (2) mice. Without exception, these areas were closely associated with localized activation of microglia (Fig. 5A_iii_ and B_iii_) and Müller glia cell (Fig. 5Aiv, B_iV_, E and F). However, no significant differences in expression of chemokine (*Ccl2*, *Ccr2*, *Cx3cl1* and *Cx3cr1*) or microglia activation marker (*iNos*, *Arg1* or *TGFb*) were detected in retinas of different *Crb1^rd8/rd8^* lines relative to wild type supporting a localized response of microglia cells to these degenerative events (Supplementary Material, Fig. S2). In all these assessments, *C57BL/6 Crb1^rd8/rd8^* (1) animals appeared similar to wild-type mice with the exception of the rare appearance of subtle aneurysm-like changes at the OPL (Fig. 5C, C_i–__iV_ and G). Three-dimensional (3D) reconstruction of large inferior lesions revealed how activated Müller and microglia cells surround several photoreceptor columns at the OPL (Fig. 5I) and contribute to the formation of gliotic scars at the OLM (Fig. 5J). These processes separate photoreceptor nuclei that have lost their orderly arrangement within the ONL from still normally packed and healthy photoreceptors in their close vicinity (Fig. 5K and L).

### Localized severe loss of photoreceptors and closely associated glial activation lead to telangiectasia-like retinal vascular remodelling

**Figure 6.**
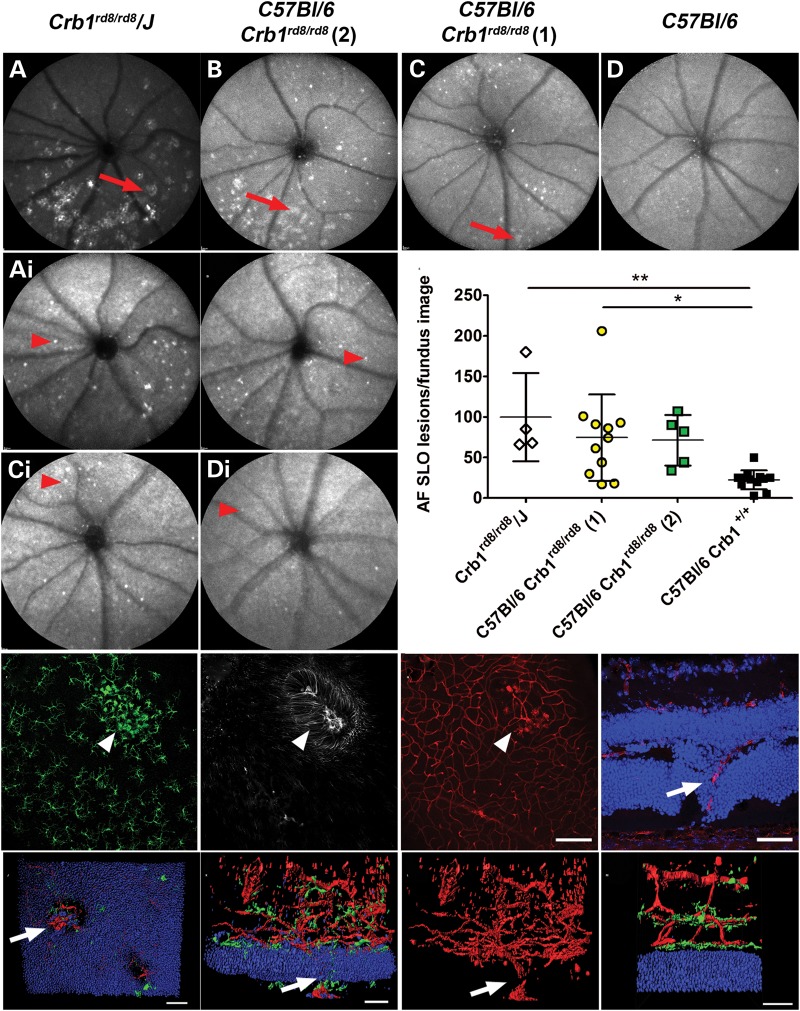
Assessment of retinal degeneration at 12 months of age and vascular remodelling at 2 and 12 months of age. (**A**–**D**) AF-SLO image focussed on the inner retina of 12-month-old mice from the respective *Crb1^rd8/rd8^* lines shows a similar degree of inferior retinal lesion as at 2 months of age indicating limited progression of the degeneration with age. (**A**_i_–**D**_i_**)** Corresponding AF-SLO images to (A)–(D), but focussed on the outer retina that revealed distinct autofluorescent spots suggestive for subretinal macrophages. (**E**) Quantification of subretinal autofluorescent spots revealed a significant increase in number of subretinal macrophages in different *Crb1^rd8/rd8^* lines compared with age-matched wild-type mice. Corresponding confocal projection image of Iba1 (**F**), GFAP (**G**) and isolectinB4 (**H**) stained retinal flat mounts at 2 months of age reveal that inside the irregular inferior retinal lesion (white arrow heads) vascular remodelling in the deep retinal capillaries occurs in close associated with Müller and microglia activation. (**I**) Retinal sections even at 2 months of age sometimes show retinal vessels that grow through the ONL towards the RPE. (**J**–**M**) 3D reconstruction of a lectinB4 labelled retinal vasculature of a *Crb1^rd8/rd8^/J* mouse at 12 months of age. (J) View from the subretinal space towards the retina revealed holes in the ONL and a retinal vessels growing through. Two-side views of the same vascular lesion represented with microglia (green) and ONL nuclei (blue) (K) or without these features (L). They also illustrate the pronounced vascular remodelling of the retinal vasculature from which the vessel extends. White arrows indicate retina-derived vessel that also grow through the RPE (Supplementary Material, movie S1). (M) Retinal vasculature with microglia (green) and ONL (blue) of an age-matched wild-type mouse shows the normal three-layer architecture of the vascular bed (Supplementary Material, movie S2).

The development of additional degenerative features during progression of the degeneration in different C*rb1^rd8/rd8^* lines was assessed by AF-SLO and OCT imaging of animals of 12 months of age and by evaluating retinal sections and flat mount preparations for aberrant vascular features using lectin B4 and CD31 labelling (Fig. 6). Mice from all lines showed at 12 months very similar inferior retinal phenotypes as at 2 months of age (Fig. 6 versus Fig. 1). The inbred *Crb1^rd8/rd8^/J* and the *C57BL/6 Crb1^rd8/rd8^* (2) lines showed again the prominent irregular lesions in the inferior retina (Fig. 6A and B, red arrows), while mice from the *C57BL/6 Crb1^rd8/rd8^* (1) line still only revealed very few small or even no inferior lesions (Fig. 6C, red arrow). However, all *Crb1^rd8/rd8^* lines showed a variable, but significant increase in additional distinct autofluorescent spots across the whole fundus indicating an accumulation of subretinal microglia/macrophages in all lines compared with age-matched wild-type mice (Fig. 6A_i_–D_i_ and E) (19). As early as 2 months, local microglia (Fig. 6F) and Müller glial activation (Fig. 6G) inside the large inferior retinal lesions were closely associated with aneurysm-like vascular structures derived from deep retinal capillaries (Fig. 6H) and with even larger vessels that extend through holes in the ONL towards the RPE (Fig. 6I, arrow). Such vessels were also seen at 12 months in severely affected *Crb1^rd8/rd8^* mice, but then extend from a strongly remodelled vascular bed (Fig. 6J–L). These vessels do not break through Bruch's membrane according to our previous analysis of this type of lesion (20). In contrast, *C57BL/6 Crb1^rd8/rd8^* (1) mice at 2 and 12 months of age did not show prominent vascular remodelling or microglia activation apart from a rare manifestation of a few small aneurysms at the OPL (Fig. 5Ci), but rather showed the three-layered vasculature typical of wild-type mice (Fig. 6M).

### Genetic loci on chromosome 15 may modify the *Crb1 ^rd8/rd8^* phenotype

Despite the phenotypic stability of the *Crb1^rd8/rd8^/J* line, and the common ancestry of the *C57BL/6 Crb1^rd8/rd8^* lines (1) and (2), there is a significant variation in the observed phenotype. We reasoned therefore that a modifying gene or genes may have been segregated between the lines. To test this, we first determined whether the different mouse lines carry any known mutant alleles commonly found in inbred mouse strains (21) that may be responsible for the modifying effect on the phenotype. We screened the mutant *Pde6b^rd1^* (c.1041C > A) allele (22), two mutant alleles in the *Gnat2* gene, *Gnat2* (c.518A>G) (23) and *Gnat2^Cpfl3^* (c.598G>A) (24), and the polymorphism in the *Rpe65 gene*, *RPE65^Met450Leu^* [*Rpe65* (c.1348C>A)], that has been previously shown to be a genetic modifier for retinal degeneration (25). None of the mutations were identified in any of the *Crb1^rd8/rd8^* lines studied (Table 1) and therefore cannot explain the observed phenotypic differences. All tested *Crb1^rd8/rd8^* lines were also homozygous for the *RPE65^Met450^* allele indicating that they all carry the allele common for the *C57BL/6* genetic background at this genomic position and thus do not differ in this respect either (Table 1).

**Table 1.** Genotyping of several mutant alleles common in inbred mouse strains did not explain the modifying effect observed in the different *Crb1^rd8/rd8^* homozygous lines

Candidate alleles	*Crb1^rd8/rd8^/J*	*C57BL/6 Crb1^rd8/rd8^(1)*	*C57BL/6 Crb1^rd8/rd8^(2)*	*C57BL/6J*	*Balb/c*	*129 S2/Sv*
*Crb1^rd8^* (c.3481delC)*	100% (10/10)	100% (7/7)	100% (15/15)	0% (0/4)	0% (0/4)	0% (0/4)
*Pde6b^rd1^* (c.1041C>A)	0% (0/10)	0% (0/7)	0% (0/15)	0% (0/4)	0% (0/4)	0% (0/4)
Gnat2 (c.518A>G)	0% (0/8)	0% (0/5)	0% (0/15)	0% (0/3)	0% (0/3)	0% (0/3)
Gnat2^Cpfl3^ (c.598G>A)	0% (0/10)	0% (0/7)	0% (0/15)	0% (0/4)	0% (0/4)	0% (0/4)
RPE65^Leu450/Leu450^ or RPE65^Met450/Met450^ (c.1348C>A)	100% (10/10) RPE65^Met450^	100% (7/7) RPE65^Met450^	100% (15/15) RPE65^Met450^	100% (4/4) RPE65^Met450^	100% (4/4) RPE65^Leu450^	100% (4/4) RPE65^Leu450^

Percentage and absolute number of the genotyping result for the respective mutant and modifying allele. These results were obtained by DNA sequencing of individual animals from the three homozygous *Crb1^rd8/rd8^/J*, *C57BL/6 Crb1^rd8/rd8^(1)* and *C57BL/6 Crb1^rd8/rd8^(2)* mouse lines (*n* = 10 each) and from wild-type animals from inbred lines with different genetic backgrounds (*C57BL/6J*, *Balb/c* and *129 S2/Sv*) that served as controls (*n* = 4 each). In addition to the represented animals in this table all animals used in this study were genotyped for the Crb1^rd8^ allele.

**Figure 7.**
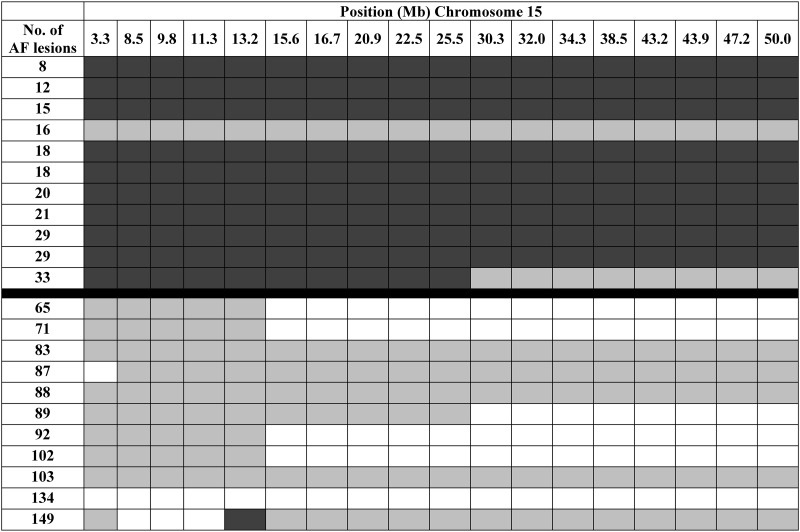
SNP genotype across a large region on chromosome 15 that shows homozygosity in mice with low number of AF-lesions (*n* = 11) (dark grey), while those with high number of AF-lesions are either heterozygous (grey) or homozygous (white) for an alternate base.

To provide further insight into a potential genetic basis for the phenotypic variability, we decided to perform DNA SNP screen analysis for animals from the different lines. The SNP data confirmed the inbred status of the *Crb1^rd8/rd8^/J* line for which we observed a higher proportion of C3H (or identical) SNPs to be present than in the two other *C57BL/6 Crb1^rd8/rd8^* lines (data not shown) suggesting that there are distinct genetic differences between the inbred *Crb1^rd8/rd8^/J* line and the two *C57BL/6 Crb1^rd8/rd8^* lines (1) and (2) that are of common ancestry. Based on this common ancestry, and the distinct phenotypic differences shown by *C57BL/6 Crb1^rd8/rd8^* line (1) and *C57BL/6 Crb1^rd8/rd8^* line (2), we decided to generate preliminary data to help identify regions in the genome that may contain modifying factors. We therefore performed an ‘analysis of extremes’ using DNA SNP screen data from animals that are from either of the two related *C57BL/6 Crb1^rd8/rd8^* lines (1) and (2), but show the most divergent (extremes) of the AF-lesion phenotype. Statistical analysis of this dataset identified a significant association between the genotype on a region of chromosome 15 and the phenotype (Table 2). As shown in Figure 7, there is a large region on chromosome 15 where mice with low AF-lesion count are homozygous for one allele (consistent with a *C57BL/6* derived allele), whereas mice with a high AF-lesion number are either heterozygous or homozygous for alternative SNPs. These data suggest that this region on chromosome 15 may carry one or several genetic modifiers that determine the manifestation of the phenotype associated with the *Crb1^rd8/rd8^* mutation.

**Table 2.** Statistical analysis of the genotyping results from both *C57/Bl6 Crb1^rd8/rd8^* lines 1 and 2 with either high (*n* = 11) or low (*n* = 11) number of autofluorescent fundus lesions

SNP	Position in bp/Chr. 15	*P*-value
rs13459176	3279130	1.01E−04
CEL-15_8331158	8474279	1.03E−04
CEL-15_9687257	9788004	6.22E−04
rs13482431	11311464	6.22E−04
rs3711814	13219494	6.22E−04
CEL-15_15482356	15577251	9.27E−05
rs13482455	16731203	9.27E−05
rs13482469	20942406	9.27E−05
rs13482477	22519205	9.27E−05
rs13482485	25450871	9.27E−05
rs13482504	30322280	2.91E−04
rs13482509	32042169	2.91E−04
rs6188239	34331233	2.91E−04
rs3695416	38484397	2.91E−04
CEL-15_43206205	43188178	2.91E−04
rs13482543	43870893	2.91E−04
rs13482558	47166660	2.91E−04
rs13482571	49998375	2.91E−04

Listed are all informative SNPs that showed significant differences between the two groups based on a Pearson's *χ*^2^ test indicating the genomic region on chromosome 15 that is associated with the incidence of the AF-lesions phenotype.

## DISCUSSION

In this study, we present data illustrating the wide range of variable retinal pathologies associated with the *Crb1^rd8/rd8^* mutation and present initial genetic data from SNP screen analyses that suggest the existence of genetic modifiers on chromosome 15 that modulate the manifestation of the *Crb1^rd8/rd8^* phenotype in mice. These findings indicate that this mutation alone is necessary, but not sufficient for the manifestation of the inferior retinal degeneration and specific associated features, which were only observed in two of the three evaluated homozygous *Crb1^rd8/rd8^* lines. This study also highlights how pronounced the influence of small genetic background differences can be on the manifestation of this retinal degeneration and presents initial data for a candidate region that may carry the underlying genetic modifier(s). This is an important issue since this mutation has been found in many mouse strains across the world (18). The present study clarifies primary and secondary confounding effects of the *Crb1^rd8/rd8^* mutation on retinal pathology in mice. Thereby, we aim to raise awareness for the variability of the confounding effects that depend on the genetic context and provide a reference for the evaluation of the current literature in the retinal field and in future experimental studies involving these mice.

### Nonsense-mediated decay may act as a primary mechanism of the rd8 allele resulting in loss of Crb1 protein and weakening of the OLM across the whole retina

In addition our data may provide new insight into the primary mechanism of action of the *Crb1^rd8/rd8^* mutation. The observed reduction of mutant *Crb1* transcripts may indicate that nonsense-mediated decay (NMD) occurs. NMD is a mechanism that results in the degradation of mRNA transcripts that carry premature stop codons and serves as a quality control to prevent the expression of deleterious mutant proteins (26,27). However, this observation does not exclude the possibility that a truncated mutant protein is expressed from the remaining 20% of *Crb1* transcripts and may still contribute to the effect of the *Crb1^rd8/rd8^* mutation by a gain of function as previously considered (14,16).

Consistent with such a null or hypomorphic effect of the rd8 allele, our data also confirm that the Crb1 protein is lost at the OLM in all lines, resulting in an evenly distributed but variable reduction of AJ at the OLM across the whole retina. However, this reduction was not fully established in each individual mouse, caused either by a sampling bias due to the analysis of sections or an intrinsic variability of the phenotype caused by line specific differences. The latter interpretation is consistent with discrepancies in the number of AJ reported for *Crb1^rd8/rd8^* and *Crb1^−/−^* mice in the literature (15,16) and further supported by the line differences in Müller cell process expansion at the OLM between the *Crb1^rd8/rd8^/J* and the *C57BL/6 Crb1^rd8/rd8^* lines observed at the ultrastructural level. Both effects correlate well in their localization, with the absence of the Crb1 protein confirming its involvement in OLM maintenance across the whole retina as previously suggested (13).

### Additional genetic modifier(s) located on chromosome 15 determine the severity and additional phenotypic features in *Crb1^rd8/rd8^* mice

Prominent differences in the manifestation of the retinal degeneration in different homozygous *Crb1^rd8/rd8^* lines and the miss-match of the localization of the degenerative phenotype with the primary OLM defect suggest that the inferior features are secondary consequences dependent on additional factors. However, the presence of the homozygous *Crb1^rd8/rd8^* mutation seems necessary since heterozygous or wild-type littermates did not show any phenotypic features when evaluated during establishment of the two *Crb1^rd8/rd8^* lines (20).

Because of the inferior nature of the degeneration, light was considered to be a good candidate for such an additional initiating factor for this type of retinal degeneration. Yet, this hypothesis was recently excluded by the observation that both, *Crb1^−/−^* and *Crb1^rd8/rd8^* mice raised in complete darkness from birth show a similar degree of inferior retinal degeneration as those raised under normal light conditions (15,20). Since all three *Crb1^rd8/rd8^* mouse lines studied here were raised in the same environment, but still showed prominent differences in their phenotype, predominantly genetic factors may define these additional phenotypic features. This also confirms a previous report, in which 19% of homozygous *Crb1^rd8/rd8^* mice on a *C57BL/6* background did not exhibit retinal spots in fundus images implicating the strong modulatory role of the genetic background (16). Our study strengthens this argument by revealing the genetic and phenotypic stability of the *Crb1^rd8/rd8^/J* inbred and the *C57BL/6 Crb1^rd8/rd8^* line (1) over multiple generations. Furthermore, our observation that the two closely related *C57BL/6 Crb1^rd8/rd8^* lines (lines 1 and 2), still differ significantly in their phenotype suggests that a few genetic factors or even a single factor may be responsible for the modifying effects in the different lines. By using an ‘analysis of extremes’ on the SNP data obtained from animals with the most divergent phenotypes from the two related *C57BL/6 Crb1^rd8/rd8^* lines (1) and (2), we identified a large region on chromosome 15 that is significantly associated with the phenotype and thus may carry such genetic modifiers.

While animals with few retinal lesions are homozygous for one allele, consistent with a *C57BL/6* allele, animals with a many lesions are either heterozygous or homozygous for an alternative allele. Therefore, the modifying factor(s) on chromosome 15 may act either as recessive suppressor(s) or as dominant enhancer(s) for the manifestation of the *Crb1^rd8^*^/rd8^ phenotype. However, our data do not allow us to conclude with certainty about the origin of the modifying factor(s) from either of the genetic backgrounds since it could be the result of a spontaneous mutation in any of the alleles. To answer these questions and gain mechanistic insight into the function of these additional genetic factors, a more extensive mapping or QTL project would be required to confirm our initial results and narrow down the genomic region on chromosome 15 which may then result in the identification of the genetic modifying factor(s) for the *Crb1^rd8/rd8^* phenotype.

### Secondary vascular remodelling in a subset of *Crb1^rd8/rd8^* lines resembles retinal telangiectasia seen in patients with *Crb1* mutations

Based on our observations, the modifying factors may be mechanistically involved in photoreceptor survival, e.g. by affecting additional genes in the *Crb1* pathway, or alter processes that define the threshold of Müller glia or microglia responses. A general innate immune activation during disease progression was indicated by the increased accumulation of subretinal macrophages in all *Crb1^rd8/rd8^* mice during later stages. Additional modulating factors that further alter such inflammatory or gliotic responses may lead to an even more permissive inflammatory and pro-angiogenic environment in the retina and may result in vascular remodelling and additional degenerative features. However, the question of why the inferior retina is preferentially affected by these processes remains unresolved. Nevertheless, it is of particular interest that the localized aneurysms- and telangiectasia-like vascular lesions in *Crb1^rd8/rd8^* mice were clinically similar to retinal telangiectasia or Coats-like vasculopathy in patients with *CRB1* mutations and that the manifestation of these lesions in humans also seems to be dependent on genetic modifying factors (6,7). Since the vascular remodelling occurred in close association with Müller and microglia activation it is tempting to speculate that activated glial cells drive this pathological process. Recent reports support such a role for tissue macrophages and Müller glia cells in regulating angiogenesis by the expression of pro-angiogenic factors such as VEGFA or by guiding endothelial cell growth and fusion during developmental and pathological angiogenesis in the retina (28–30). Our data now present a starting point for the design of future genetic studies that aim to identify these modifying factors. It not only has provided evidence that the number of lesions in the inferior retina of *Crb1^rd8/rd8^* mice may be useful as a quantitative trait for genetic studies (31) but also presents a candidate region on chromosome 15 that—if confirmed—needs to be narrowed down in order to identify the modifying factor(s). These factors may not only be of particular relevance to establish genotype–phenotype correlations for retinal degenerations caused by mutation in *CRB1*, but may also help to identify novel pathways that could be targeted therapeutically for the modulation of inflammatory neurovascular processes contributing to the progression of inherited and multifactorial retinal degenerations.

## MATERIALS AND METHODS

### Animals

A *Crb1^rd8/rd8^/J* inbred line derived from the Jackson laboratory (#003392; referred to as *Crb1^rd8/rd8^/J*) was compared with two genetically related homozygous *Crb1^rd8/rd8^* lines obtained from a backcross experiment with *C57BL/6J Ola Hsd* mice, referred to as *C57BL/6J Crb1^rd8/rd8^* (1) and *C57BL/6J Crb1^rd8/rd8^* (2), respectively (20). Genotyping for the *Crb1^rd8^* allele and other mutant alleles was performed as described previously using polymerase chain reaction (PCR) amplification with flanking primers and subsequent sequencing (20). The genomic PCR primer and the respective mutant alleles are shown in Table 3.

**Table 3.** Primer pairs used for PCR amplification on genomic DNA and subsequent sequencing for genotyping of mutant or modifying alleles

Allele	Primer sequences	References
Crb1^rd8^ (c.3481delC)	Forw: gcacaatagagattggaggc Rev: tgtctacatccacctcacag	(20)
Gnat2^Cpfl3^ (c.598G>A)	Forw: catcgagaccaagttttctg Rev: accatgtcgtaggcactgag	(24)
Gnat2 (c.518A>G)	Forw: accgatgccaccttcttttt Rev: tgctgtgagacctgagatgc	(23)
Pde6b^rd1^ (c.1041C>A)	Forw: cacacccccggctgatcactg Rev: ctgaaagttgaacatttcatc	(22)
RPE65^Leu450/Leu450^ or RPE65^Met450/Met450^ (c.1348C>A)	Forw: tgacaaggtaataaagcatc Rev: attaccatcatcttcttcca	(25)

Mice were anaesthetized by intraperitoneal injection of medetomidine hydrochloride (1 mg/kg body weight; Domitor; Pfizer Animal Health, New York, NY, USA) and ketamine (60 mg/kg body weight). Pupils were dilated with 1% tropicamide. Animal experiments were performed in accordance with the ARVO Statement for the Use of Animals in Ophthalmic and Vision Research and the UK Home Office licence (PPL 70/1279).

### Housing

To minimize environmental differences, all lines were housed under the same 12 h/12 h dark/light cycling. During light, animals were exposed to an illuminance of 38 ± 28 lux (mean ± SD; range 7–100 lux), when lids, food and water bottles were in place. Outside the cages 200 ± 136 lux (range 50–500 lux) were measured (32).

### AF-SLO, OCT and TEFI imaging for *in vivo* phenotyping

As described previously, autofluorescent fundus images and OCT images were obtained using the HRA2 scanning laser ophthalmoscope with a 55° angle lens and the Spectralis™ HRA+OCT with a 30° angle lens, respectively (Heidelberg Engineering, Heidelberg, Germany) (20). The optic disc was positioned at the centre and 30 frames for AF-SLO or 50 frames for OCT images were acquired. Colour fundus images were taken by TEFI using Viscotears (Novartis Pharmaceuticals, UK) as a coupling agent with a 5 cm endoscope of 3 mm outer diameter (1218AA; Karl Storz, Tuttlingen, Germany) connected to a Nikon D300 s camera (33). All images were processed with Adobe Photoshop CS2 (Adobe Systems Incorporated, San Jose, CA, USA).

### Quantification of autofluorescent AF-SLO images

The severity of the phenotype (all autofluorescent signals) and the number of subretinal macrophages (distinct small spots) were determined by counting respective lesions on images focussed on the superficial retina for pathology or on the outer retina for subretinal macrophages.

### Histopathology and ultrastructural assessment of adhesion plaques

Semithin and ultrastructural histology of superior to inferior oriented sagittal sections of the retina were performed as previously described (19,32). After cardiac perfusion with 1% paraformaldehyde (PFA), eyes were post-fixed in 3% glutaraldehyde and 1% PFA in 0.08 m sodium cacodylate–HCl (pH 7.4) and processed for embedding in araldite resin (Agar Scientific Limited, Essex, UK). Semithin (0.7 µm) and ultrathin sections (70 nm) were stained accordingly and imaged by bright field microscopy (Oberserver.Z1 Axio, Carl Zeiss Microimaging, Jena, Germany) or transmission electron microscopy (TEM) with a JEOL 1010 connected to a Gatan Orius CCD camera. Calibrated images were imported into Image J for quantification (34). Electron dense adhesion plaques were counted in five linearly stitched TEM images at ×1000 magnification and normalized to the distance counted.

### Immunohistochemistry and 3D reconstruction

Tissue was fixed and dissected in 4% PFA, cryoprotected with 20% sucrose and embedded in OCT (Tissue Tek, Sakura Finetek, Thatcham, UK). Eighteen micrometres of retinal sections or flat mounts were blocked with 1% BSA (Sigma-Aldrich, Steinheim, Germany)/5% goat serum (AbD Serotec, Kidlington, UK) including 3% (flat mounts) or 0.3% (sections) Triton X-100 for permeabilization followed by overnight incubation at 4°C with respective primary and secondary antibodies (Table 4). The ApopTag^®^ *In Situ* Apoptosis Detection kit was used for TUNEL labelling according to manufacturer's instruction (Millipore, Watford, UK). After counterstaining with Hoechst 33342 (Dako, Cambridgeshire, UK) and mounting in fluorescent mounting medium (Dako), images were taken by confocal microscopy (Leica DM5500 Q, Leica Microsystems, Wetzlar, Germany). High-resolution Z-stack images were used for 3D reconstruction with Imaris (Bitplane, Zurich, Switzerland).

**Table 4.** Primary and secondary antibodies used in this study with respective working concentrations

Primary and secondary antibodies/reagent	Company (product code/clone)	Working dilution (final concentration)
Isolectin B_4_ (BSI-B_4_)	Sigma (L2140)	1 : 200 (5 µg/ml)
Rabbit anti-Iba1 antibody	WAKO (No. 019-19741)	1 : 500 (1 µg/ml)
Rat anti-GFAP	Merck Chemicals (# 345860/2.2B10)	1 : 200 (2.5 µg/ml)
Rabbit anti-ZO-1	Abcam (Ab59720)	1 : 200 (0.5 µg/ml)
Rabbit anti-Crb1	Gift from Jan Wijnholds	1 : 200
goat anti-rabbit AlexaFluor 488 nm-conjugated highly cross adsorbed	Life Technologies Ltd #A11034	1 : 500 (4 µg/ml)
Alexa Fluor^®^ 488 goat anti-rat IgG (H+L)	Life Technologies Ltd #A11006	1 : 500 (4 µg/ml)
Alexa Fluor^®^ 546 goat anti-rabbit IgG (H+L) highly cross adsorbed	Life Technologies Ltd #A11035	1 : 500 (4 µg/ml)
Alexa Fluor^®^ 546 goat anti-rat IgG (H+L)	Life Technologies Ltd #A11081	1 : 500 (4 µg/ml)
streptavidin, Alexa Fluor 633 conjugate	Life Technologies Ltd #S21375	1 : 500 (2 µg/ml)

### Genomic SNP screening and ‘analysis of extremes’

SNP screening was performed by the Mammalian Genetics Unit at the MRC Harwell, Oxfordshire, UK. Twenty microliters of genomic DNA (50 ng/µl in TE) per animal were analysed on an SNP array containing 1449 allele-specific probes from across the genome distinguishing *C57BL/6* and *C3H* alleles. To assess whether the observed SNP variant between the two groups and the two background strains was purely random, a *χ*^2^ test was applied. For comparison between the two groups, a frequency tables were generated by grouping animals by the base variants seen in each strain. A Pearson's *χ*^2^ test was then used to determine whether the variant frequencies differed purely by random or were significantly associated with the phenotype.

### Quantitative reverse transcriptase-polymerase chain reaction

Total RNA from retinas was extracted using the RNeasy Mini Kit (Qiagen, Manchester, UK) followed by cDNA synthesis with the QuantiTect Reverse Transcription kit (Qiagen). For Real Time PCR analysis, 20 μl reactions were prepared in triplicates containing the FastStart TaqMan Probe Master Mix (Roche, West Sussex, UK) with 5 µl of template cDNA, 200 nm of each primer and 100 nm of the corresponding FAM-labelled probe (see Table 5). Assays were run in 96-well plates using an ABI PRISM™ 7900HT Fast Real Time PCR System (Applied Biosystems, Paisley, UK) at default settings. Data were analysed using the SDS 2.2.2 software (Applied Biosystems).

**Table 5.** Real-time PCR assays with corresponding primers and Roche FAM-labelled universal probes

Assay	Primer sequences	Roche universal probe library
*Ccl2*	Forw: catccacgtgttggctca Rev: gatcatcttgctggtgaatgagt	#62
*Ccr2*	Forw: acctgtaaatgccatgcaagt Rev: tgtcttccatttcctttgatttg	#27
*Crb1*	Forw: caactcagcccatgtcctc Rev: aaaacagcctttgcgataca	#32
*Crb2*	Forw: caggattctctggccagttc Rev: caggcactgctacctccag	#34
*Cx3cl1*	Forw: ccgaattcctgcactcca Rev: catgatttcgcatttcgtca	#66
*Cx3cr1*	Forw: ggagtctgcgtgagactgg Rev: agcagatgggaagggaactt	#4
*Nos2*	Forw: ctttgccacggacgagac Rev: tcattgtactctgagggctgac	#13
*Arg1*	Forw: gaatctgcatgggcaacc Rev: gaatcctggtacatctgggaac	#2
*Tgfb1*	Forw: tcagacattcgggaagcagt Rev: acgccaggaattgttgctat	#56
Actb	Forw: aaggccaaccgtgaaaagat Rev: gtcgtacgaccagaggcatac	#56

Assays were designed using the online Assay Design Centre (www.roche-applied-science.com).

### Statistical analysis

Statistical analyses were performed using GraphPad Prism 5 (GraphPad Software, Inc., La Jolla, CA, USA).

## SUPPLEMENTARY MATERIAL

Supplementary Material is available at *HMG* online.

## FUNDING

R.R.A. is partly funded by the Department of Health's National Institute for Health Research (NIHR) Biomedical Research Centre at Moorfields Eye Hospital and Alcon Research Institute. J.W.B.B. is supported by an NIHR Research professorship, C.J.C. is a joint MRC and Fight for Sight Clinical Research Training Fellow (G1100383), P.H. is supported by the German Research Foundation (DFG-He 6175/1-1). S.-M.k.H. is partly supported by the European Union Seventh Framework Programme (FP7/2007-2013) under grant agreement n° 281234, the Medical Research Council (core support at LMCB) and the Batten Disease Family Association, UK. Funding to pay the Open Access publication charges for this article was provided by RCUK.

## Supplementary Material

Supplementary Data
