# Correction to: The severity of retinal pathology in homozygous Crb1*^rd8/rd8^* mice is dependent on additional genetic factors

**DOI:** 10.1093/hmg/ddaf191

**Published:** 2025-12-17

This is a correction to Ulrich F.O. Luhmann, Livia S. Carvalho, Sophia-Martha kleine Holthaus, Jill A. Cowing, Simon Greenaway, Colin J. Chu, Philipp Herrmann, Alexander J. Smith, Peter M.G. Munro, Paul Potter, James W.B. Bainbridge, and Robin R. Ali, The severity of retinal pathology in homozygous Crb1*^rd8/rd8^* mice is dependent on additional genetic factors, *Human Molecular Genetics,* Volume 24, Issue 1, 1 January 2015, Pages 128–141, https://doi.org/10.1093/hmg/ddu424.

In December 2024, a reader contacted the journal about concerns regarding Figure 1B and 6Di. These concerns were also raised on PubPeer (see https://pubpeer.com/publications/C44D763414B6F9DB6634DE36AFEED8). The journal contacted all co-authors in line with COPE guidance.

After reviewing their raw data, the authors determined that the image to illustrate the control C57/Bl6 mouse phenotype (i.e., the normal wild-type mouse) at 8 weeks in Figure 1B was previously published in Figure 2A in Luhmann et al. in *PloS One* (1). Also, the image to illustrate the control C57/Bl6 mouse phenotype at 12 weeks in Figure 6Di was previously published in Figure 1A in Luhmann et al. in *Experimental Eye Research* (2). This reuse of images was not disclosed in the article.

The authors requested a correction to the Methods section describing the Animals used in the study. The corrected paragraph is as follows: A *Crb1rd8/rd8/J* inbred line derived from the Jackson laboratory (#003392; referred to as *Crb1rd8/rd8/J*) was compared with two genetically related homozygous *Crb1rd8/rd8* lines obtained from a backcross experiment with *C57BL/6J Ola Hsd* mice, referred to as *C57BL/6J Crb1rd8/rd8* (1) and *C57BL/6J Crb1rd8/rd8* (2), respectively (20). Phenotypic and genetic data utilized in this study were obtained from as many animals as possible, including from animals contributing to prior publications addressing different scientific objectives. (20, 32). Genotyping for the *Crb1rd8* allele and other mutant alleles was performed as described previously using polymerase chain reaction (PCR) amplification with flanking primers and subsequent sequencing (20). The genomic PCR primer and the respective mutant alleles are shown in Table 3.

The articles in which the images were previously published were already cited within the article, and this correction does not change the order of the references.

In addition, the figure legend for Figure 1 should end with this additional sentence: The image for *C57Bl/6 Crb1+/+* in panel B is reproduced from Luhmann et al. [*C57Bl/6* in Figure 2A (20)] under the terms of the Creative Commons Attribution License. The figure legend for Figure 6 should end with this following sentence: the image in panel 6Di is reproduced from Luhmann et al. [*C57Bl/6* in Figure 1A (23)] under the terms of the Creative Commons Attribution License.

The Editors have carefully reviewed the correction to the Methods section and are satisfied that this correction does not alter the conclusions reached in the original article.

These details have been corrected only in this correction notice to preserve the published version of record.
