# Supplementary material for: The severity of retinal pathology in homozygous Crb1rd8/rd8 mice is dependent on additional genetic factors
Source: Hum Mol Genet. 2014 Aug 21;24(1):128–41. doi: 10.1093/hmg/ddu424 (PMC4262495; doi:10.1093/hmg/ddu424)
Supplement: Supplementary Data [file supp_24_1_128__index.html]

The severity of retinal pathology in homozygous Crb1rd8/rd8 mice is dependent on additional genetic factors — The severity of retinal pathology in homozygous Crb1rd8/rd8 mice is dependent on additional genetic factors — The severity of retinal pathology in homozygous Crb1rd8/rd8 mice is dependent on additional genetic factors — Supplementary Data 

# The severity of retinal pathology in homozygous *Crb1rd8/rd8* mice is dependent on additional genetic factors

## Supplementary Data

Supplementary Data

**Files in this Data Supplement:**

- Supplementary Data - Doc file
- Supplementary Video 1 - avi file
- Supplementary Video 2 - avi file
