# Supplementary material for: The severity of retinal pathology in homozygous Crb1rd8/rd8 mice is dependent on additional genetic factors
Source: Hum Mol Genet. 2014 Aug 21;24(1):128–41. doi: 10.1093/hmg/ddu424 (PMC4262495; doi:10.1093/hmg/ddu424)

**Supplemental Figure 1: Comparison of expression levels of *Crb1* in the retina of wildtype mice on different genetic backgrounds with a mouse line carrying the *Crb1^rd8/rd8^* mutation.**

Relative quantitative RT-PCR analysis of *Crb1* RNA levels in retinas of *C57BL/6*, *Balb/c*, *Sv129* and *Crb1^rd8/rd8^/J* mice at 8 weeks of age (n = 4 each). As indicated by the significant reduction of *Crb1* expression in *Balb/c* mice (0.47 ± 0.16 %) compared to C57BL/6 mice (1 ± 0.22) and by the intermediate expression level of *Crb1* in retinas of *Sv129* mice (0.71 ± 0.14 %), different genetic backgrounds can influence expression levels of *Crb1* in the retina. However compared to the genetic background influence on *Crb1* expression, the reduction in *Crb1* expression due to the *Crb1^rd8/rd8^* mutation is always more pronounced in *Crb1^rd8/rd8^/J* mice compared to all three other wildtype strains. All samples were normalized to levels of β-actin and are presented relative to the average level of *Crb1* in C57BL/6 retina. * (p<0.05), **, (p<0.01), *** (p<0.001) indicate significant differences base on Oneway ANOVA with Tukey’s multiple comparison analysis.

**Supplemental Figure 2: Relative quantitative Real time PCR measurements of levels of chemokine RNA and microglia activation marker in retinae of wildtype and *Crb1^rd8/rd8^* mice with different degree of degeneration.** No significant alterations in RNA levels were observed neither for the chemokine ligand receptor pairs of Ccl2 (A) - Ccr2 (B) and Cx3cl1 (C) and Cx3cr1 (D) nor for the microglia activation marker iNOS (E), Arg1 (F) and TGFβ (G). All samples were normalized to levels of β actin and are presented relative to the average level of each transcript in wildtype retina.

**Supplemental movies 1 & 2**: 3D Imaris reconstruction of the retinal vasculature to illustrate remodelling of the retinal vascular beds and a single telangiectasia like lesion in a *Crb1^rd8/rd8^/J* mouse at 12 months of age (supplemental movie 1) and a *C57BL/6* mouse for comparison (supplemental movie 2). Endothelial cells are shown red, microglia in green and cell nuclei of only the ONL and the RPE in blue.

**FIG S1**


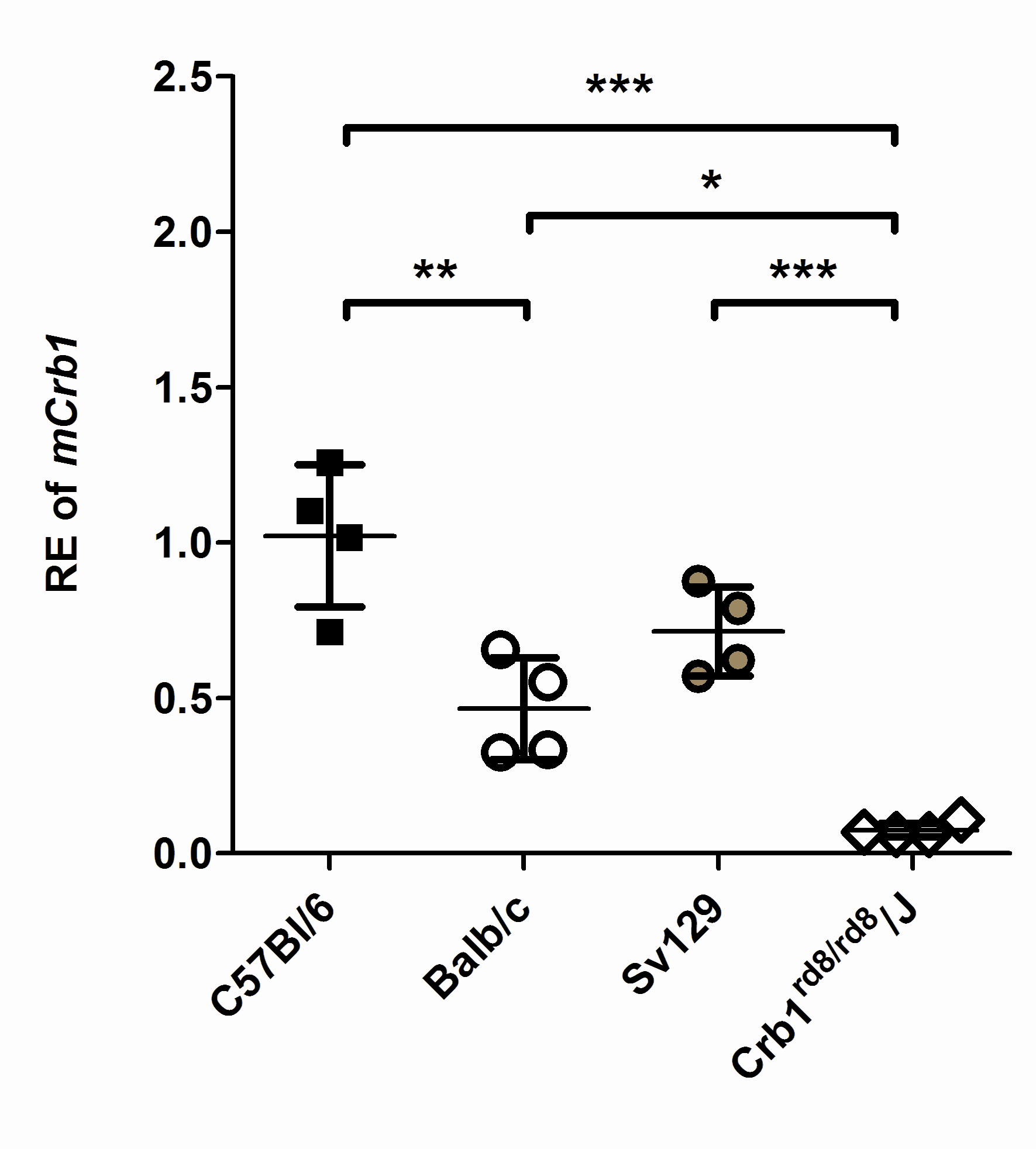


**FIG S2**


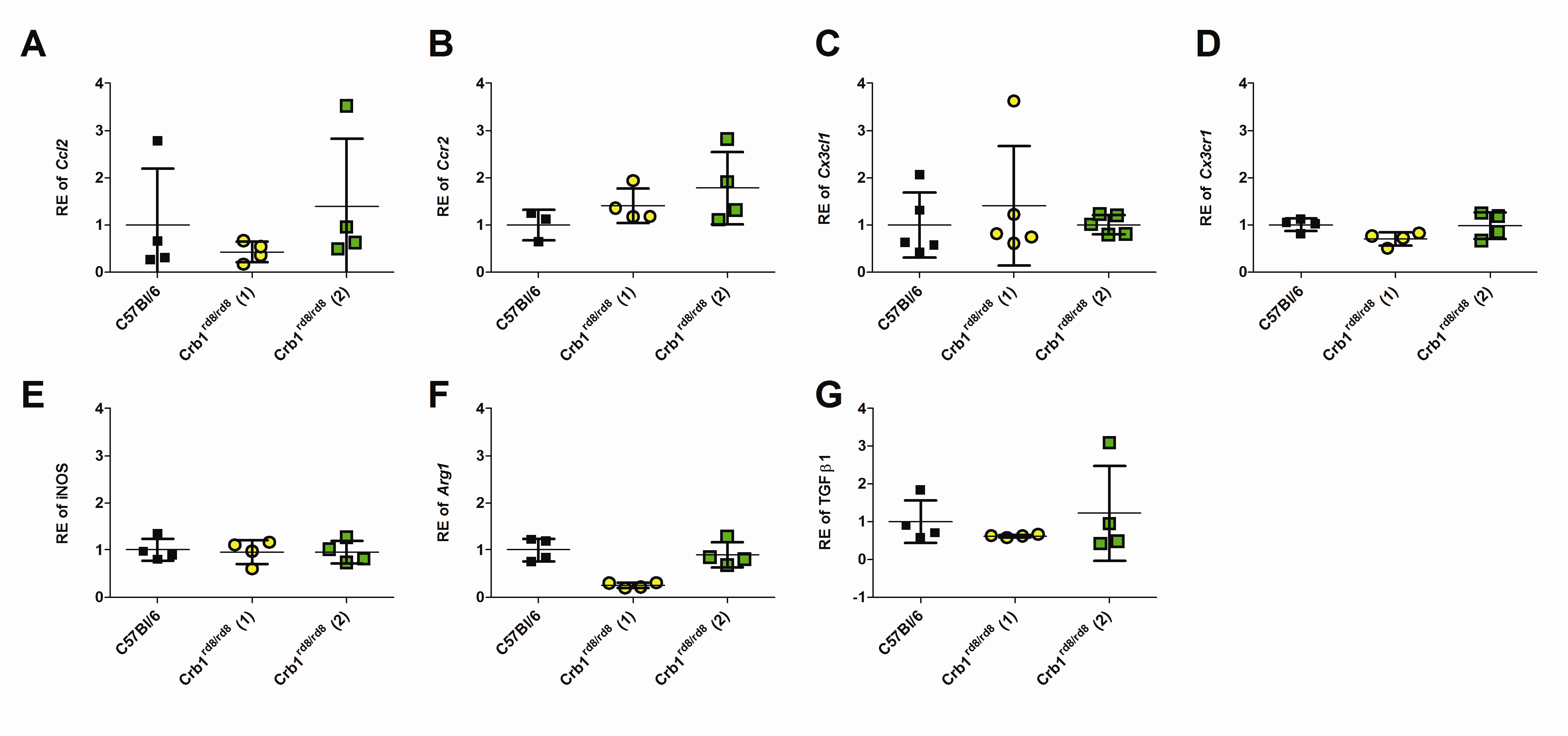

Supplement: Supplementary Data [file supp_ddu424_ddu424supp.doc]
